# Diagnostic Approaches for COVID-19 and Its Associated Complications

**DOI:** 10.3390/diagnostics11112071

**Published:** 2021-11-09

**Authors:** Ivan E. Wang, Grant Cooper, Shaker A. Mousa

**Affiliations:** The Pharmaceutical Research Institute, Albany College of Pharmacy and Health Sciences, 1 Discovery Drive, Rensselaer, NY 12144, USA; Ivan.Wang@acphs.edu (I.E.W.); Grant.Cooper@acphs.edu (G.C.)

**Keywords:** COVID-19 and complications, SARS-CoV-2 infection, COVID-19 sampling and testing, CRIPSR technology in COVID-19 diagnosis

## Abstract

With almost 4 million deaths worldwide from the COVID-19 pandemic, the efficient and accurate diagnosis and identification of COVID-19-related complications are more important than ever. Scales such as the pneumonia severity index, or CURB-65, help doctors determine who should be admitted to the hospital or the intensive care unit. To properly treat and manage admitted patients, standardized sampling protocols and methods are required for COVID-19 patients. Using PubMed, relevant articles since March 2020 on COVID-19 diagnosis and its complications were analyzed. Patients with COVID-19 had elevated D-dimer, thrombomodulin, and initial factor V elevation followed by decreased factor V and factor VII and elevated IL-6, lactate dehydrogenase, and c-reactive protein, which indicated coagulopathy and possible cytokine storm. Patients with hypertension, newly diagnosed diabetes, obesity, or advanced age were at increased risk for mortality. Elevated BUN, AST, and ALT in severe COVID-19 patients was associated with acute kidney injury or other organ damage. The gold standard for screening COVID-19 is reverse transcriptase polymerase chain reaction (RT-PCR) using sputum, oropharyngeal, or nasopharyngeal routes. However, due to the low turnover rate and limited testing capacity of RT-PCR, alternative diagnostic tools such as CT-scan and serological testing (IgM and IgG) can be considered in conjunction with symptom monitoring. Advancements in CRISPR technology have also allowed the use of alternative COVID-19 testing, but unfortunately, these technologies are still under FDA review and cannot be used in patients. Nonetheless, increased turnover rates and testing capacity allow for a bright future in COVID-19 diagnosis.

## 1. Introduction

On 31 December 2019, the World Health Organization announced to the world that a “viral pneumonia of unknown cause” reported in Wuhan, People’s Republic of China, can infect humans [1]. It was first termed a novel coronavirus strain (later named by the International Committee of Taxonomy of Viruses as Severe Acute Respiratory Syndrome Coronavirus 2, SARS-CoV-2) and is responsible for Coronavirus Disease 2019 (COVID-19). With almost 4 million deaths worldwide, the prevention, diagnosis, and treatment of the disease and its complications and the research and development in vaccines are more important than ever. The focus of this review article is the direct diagnostic approach for COVID-19 and its associated complications.

Symptoms associated with COVID-19 can be unspecific but often include fever, dry cough, and tiredness. Less common symptoms can include aches and pains, nasal congestion, headaches, conjunctivitis, gastrointestinal discomfort, loss of senses such as taste and smell, lymphopenia, and prolonged symptomatic experience (more than 3 days) with antimicrobial treatment [2]. COVID-19 has also been associated with abnormalities such as immune deficiency; coagulation abnormalities; myocardial, hepatic, and kidney injury; and secondary bacterial infection [3]. Although most infected individuals do not require intervention or hospitalization, around one in every five patients develop serious symptoms such as difficulty breathing. Individuals with comorbid disease states such as high blood pressure, heart and lung diseases, diabetes, or cancer are at increased risk for developing complications associated with SARS-CoV-2 infection.

In an effort to reduce the transmission of the virus, patients who are at increased risk should be sampled by personnel trained with proper biosafety instructions and wearing personal protective equipment including a face shield, face mask, gloves, and have used proper hand sanitation using hand soap for at least 20 s or with an alcohol-based hand sanitizer (at least 60% alcohol) [4]. Currently, patients who are hospitalized with or without symptoms and health care facility workers with or without symptoms should receive testing for SARS-CoV-2 [5,6]. Additionally, individuals who are from racial or ethnic minority groups such as African Americans, Hispanics and Latinos, and Native American tribes should receive testing due to the association of worsened COVID-19 outcomes [5,6,7]. The goal is for individuals who are at risk due to health status or socioeconomic status to be tested because SARS-CoV-2 can be transmitted by individuals who are asymptomatic or during the latency period (3–7 days, with a maximum of 14 days) [2,8]. 

With the 2020–2021 winter flu season and the possibility of annual recurrence, the demand for SARS-CoV-2 testing will likely increase, and there is a need for high-quality diagnostic tests with fast turnover times. Modeling established viral diagnostic tests such as those for human immunodeficiency virus (HIV), an ideal diagnostic test for COVID-19 should be 100% sensitive and specific, inexpensive, easy to train individuals to use, and can be used without too much clinical experience [9]. Additionally, for optimal usability the diagnostic test should be able to detect viral particles both early in infection (3–7 days post infection) as well as late (20 to 37 days) in the infection when the viral load is lower [10]. Although the current standard diagnostic tool, reverse transcriptase polymerase chain reaction (RT-PCR), requires the samples to be refrigerated, an ideal test would not have such restrictions so as to optimize its use in developing nations with warm climates that often lack the cold-chain infrastructure for proper sample transport. In addition, the diagnostic test would need to be sensitive for emerging SARS-CoV-2 variants. 

While the global spotlight is currently focused on combating this pandemic through means ranging from finding a treatment among existing therapeutic agents to development of vaccines that can help in overcoming complications against the different variants. However, diagnostics and vaccine development are inseparable, and those key aspects are what this review dealt with. Such an example for the value of early diagnosis is to identify subjects at risk, and the quick initiation of the treatment may help to provide patients with a more favorable outcome in rare cases of vaccine-induced immune thrombotic thrombocytopenia and thrombosis after viral vector vaccines. 

### 1.1. Structure and Etiology of Coronavirus

First identified under an electron microscope, the morphology of the coronavirus is spherical with corona-like projections consisting of a matrix of glycoproteins. These glycoproteins consist of spike protein S with the overall virus ranging from a size of 70 to 120 nm in diameter [11,12]. Coronaviruses are single-stranded (positive-sense) RNA viruses that code for the replicase-transcriptase spike, an enveloped membrane and a nucleocapsid protein [12]. SARS-CoV-1 virus, Middle East Respiratory Syndrome coronavirus (MERS-CoV) virus, and the current SARS-CoV-2 virus fall under the subfamily of Coronavirinae in the order Nidovirales and often cause respiratory-like symptoms. 

Using a genome search of the SARS-CoV-1 virus responsible for the SARS outbreak in 2003, the MERS-CoV virus responsible for the MERS outbreak in 2012, the SARS-CoV-2 virus responsible for the COVID-19 pandemic, and other coronaviruses isolates in bats, researchers Lu and colleagues showed that SARS-CoV-2 was 88% identical to two bat-derived SARS-like coronaviruses collected in 2018 in Zhoushan, China but more distant from SARS-CoV-1 (~79% identical) and MERS-CoV (~50% identical) [13]. These early findings show that not only do viruses mutate, which provides a challenge in diagnosis, treatment, and vaccine development, but that SARS-CoV-2 is a novel virus that has evolved and adapted from previous treatments, making virus diagnosis, its ability to mutate, and subsequent complications more troublesome. 

Similar to SARS-CoV-1, SARS-CoV-2 first binds to angiotensin-converting enzyme-2 (ACE2) through the spike protein. Interestingly, SARS-CoV-2 binding to ACE2 has been shown to be of higher affinity as compared with SARS-CoV-1 [3,14]. The spike protein must be primed by a protease, which is TMPRSS2. Once primed, the virus enters the host cell, inserts its genome into the host genome, and replicates through host genome replication [3]. The insertion of SARS-CoV-2 into the host genome allows the detection of infection though methods such as RT-PCR. 

### 1.2. Severity and Subsequent Need for Hospitalization

The severity of COVID-19 can be determined by considering symptoms in combination with serology. In patients with COVID-19 who develop pneumonia, scales such as the pneumonia severity index (PSI) shown in Table 1 and CURB-65 (acute Confusion, Urea concentrations, Respiratory rate, Blood pressure, and age greater than 65) shown in Table 2 can be used to assess the severity and subsequent need for hospitalization [15]. Once admitted to the hospital, the requirement for intensive care unit (ICU) admission is dependent on the American Thoracic Society’s and the Infectious Disease Society of America’s clinical guidelines [16,17]. Patients who have one major criterion or three minor criteria should be admitted to the ICU. Major criteria include the need for invasive mechanical ventilation and hypotensive shock with the need for vasopressors. Minor criteria include a respiratory rate greater than 30 respirations per minute, partial pressure of oxygen in blood over fraction of inhaled oxygen of (PaO2/FiO2) less than 250, multi-lobar infiltrates, confusion or disorientation, uremia (Blood Urea Nitrogen (BUN) > 20 mg/dL), leukopenia, thrombocytopenia (platelets < 100,000 cm/mm^3^), hypothermia (temperature < 98.2 °F), and hypotension requiring aggressive fluid administration [18]. A recent study noted that patients with underlying cardiovascular conditions should also be admitted to the ICU, if available, due to the risk of developing severe disease status such as chest tightness, fatigue, and myalgia (odds ratio (OR) 2.652) [19]. 

### 1.3. Sampling 

In patients who do not require hospitalization, sampling can be completed using a nasopharyngeal swab or an oropharyngeal swab. The swabs should be placed and transported in the same tube with a viral transport medium for analysis using RT-PCR. In patients with symptoms of more severe disease or who develop pneumonia, either bronchoalveolar lavage or tracheal aspirate are the recommended sampling methods. According to the COVID-19 laboratory testing guideline issued by the National Health Commission of China, fecal specimens (10 g), anal swabs, blood specimens (5 mL), or serum specimens are also possible alternatives for COVID-19 sampling. The collected specimen should be tested as soon as possible but can be stored at 4 °C for up to 24 h or at −70 °C. For serum, storage at 4 °C for up to 3 days is also possible [20]. 

The Centers for Disease Control (CDC) states that the lowest detectable concentration for SARS-CoV-2 using RT-PCR with 95% confidence (where 19/20 samples test positive) is 100.5 RNA copies/μL for the QIAGEN EZ1 Advance XL method and 1 RNA copy/μL using the QIAGEN DSP Viral RNA Mini Kit [21]. Bara et al. demonstrated that the specificity of the SARS-CoV-2 N1 (N1) primer is 100% with a limit of detection of 21 copies/reaction [22]. The SARS-CoV-2 N2 (N2) primer has also been demonstrated to be less specific and sensitive when compared to N1, however, the amount is not currently known. N3 has been removed from the CDC’s approved test due to the lack of sensitivity and specificity [22,23]. These limits reinforce the importance of sampling patients.

The CDC states that a valid RT-PCR test must have three controls, a “No Template Control” (which measures if contamination has taken place and should test negative for N1 and N2 and RNase P (RP) primer), a “2019-nCoV Positive control” (which will test positive with the correct primer and probe set used: N1, N2 and RP), and a “Human Specimen Control” (which determines if nucleic acid extraction was successful and should only test positive for the RP). With a fluorescent probe, extraction using the RNase P reaction must have a growth curve that crosses the threshold of 40 cycles, which indicates a positive RNase P gene, a human control. If the threshold is not reached, the results are invalid [21].

If the sample tests are positive, serological test can further confirm the immune response to SARS-CoV-2 infection. According to the CDC, individuals who test positive should be quarantined until symptom resolution and a possible negative laboratory test [24]. However, this is not always the case, as there have been numerous circumstances where individuals still test positive after symptom resolution. Recent studies have shown that viral shedding continued to happen in positive patients 17 to 20 days from the resolution of symptoms [10]. Newer recommendations suggest that patients who have low viral loads can be considered non-contagious and return into society [25]. This, again, reinforces the importance of using diagnostic and serological tests to accurately measure viral particles in late stages of the disease. 

## 2. Materials and Methodology

Using PubMed, articles published within the last 7 months (February to September 2020) that reported diagnostic procedures or techniques for the identification of SARS-CoV-2 or COVID-19 were analyzed. The included articles were limited to clinical trials and randomized controlled trials. Using keyword searching, terms such as “COVID-19”, “novel coronavirus”, “coronavirus”, “SARS-CoV-2”, “diagnosis”, and “complications” were used to generate a preliminary list of literature. Due to the abundance of literature, subsequent literature searches were completed using keywords such as “D-dimer”, “cytokine”, “cytokine storm”, “coagulation factor”, “coagulopathy”, “RT-PCR”, “Chest CT-ray”, “serological testing”, or “CRISPR technology” to obtain more focused articles.

## 3. Diagnostics and Complications

### 3.1. D-dimer, Coagulation Factors, and Troponin

In most published articles on patients that tested positive for SARS-CoV-2, D-dimer was the most prominent factor to determine COVID-19 severity and subsequent complications (Table 3). Liu et al. found that the optimal threshold for D-dimer is 1.56 μg/mL, with D-dimer levels above 1.56 μg/mL leading to increased mortality (n = 336) [26]. Because D-dimer is a marker for an activated coagulation cascade, many researchers looked at the risk factors associated with COVID-19 and for D-dimer to predict worsened health outcomes. Generally, patients who were hospitalized and, in the ICU, had the highest concentrations of D-dimer, followed by hospitalized patients and outpatient individuals [27]. This correlated with increased blood coagulation disorders with diagnosed COVID-19 [28]. 

D-dimer was shown to lead to increased thrombi generation and fibrinolysis with clots that were firmer than in COVID-19-negative patients. Increased prophylaxis for thrombosis using low molecular weight heparin or unfractionated heparin with a direct thrombin inhibitor was beneficial in preventing complications with thrombosis [29]. Additionally, Demelo-Rodriguez et al. noted that a D-dimer cutoff of 1.5 μg/mL had an 85% sensitivity and an 88.5% specificity in detecting venous thromboembolism (VTE) in patients (n = 156) with COVID-19 in a hospital [30]. Another study by Al-Samkari and researchers showed that a D-dimer level of 1–2.5 μg/mL corresponded to an OR of 3.04 and that a D-dimer level of > 2 μg/mL led to an OR of 6.79, showing that an increase in D-dimer concentration correlated with a further increased risk for developing thrombosis [31]. Although more trials are needed to make a recommendation on a target D-dimer level for complications, as well as to determine if the bleeding risk is a result of increased inflammation, studies showed that D-dimer levels as high as 1 μg/mL led to increased bleeding or thrombosis complications, which are linked to critical illness and death [10]. 

In addition to D-dimer, there are also other coagulation markers that are dysregulated such as factor V, factor VII, factor VIII, and von Willebrand factor (VWF) in patients with COVID-19 and who are at risk for VTE (Table 3). In a clinical trial conducted by Goshua and colleagues on 68 hospitalized patients, 80% had elevated the VWF antigen, 75% had elevated the VWF activity, and 90% had elevated factor VIII, all of which were above the normal range [32]. Patients in the ICU had even higher levels of VWF activities that were above the limit of detection. Although the increase in thrombomodulin (a marker for endothelial cell function) was not significant, VWF and thrombomodulin were correlated with increased mortality in these patients. Patients (88%) having low soluble thrombomodulin (<3.26 ng/mL) were discharged from the hospital, whereas only 52% of patients having high soluble thrombomodulin (>3.26 ng/mL) were discharged, showing that low soluble thrombomodulin correlated with improved in-hospital mortality [32]. In a study by Zhang and colleagues, factor VIII was shown to be 307% above the normal range, and factors V and VII were lower in near-terminal stage patients (n = 19) [33]. This showed a contrast to the initial elevated factor V levels found by Stefely and researchers at the initial onset of SARS-CoV-2 infection [33,34]. Although patients (n = 102) with elevated factor V (>150 IU/dL) initially had a higher incidence of deep vein thrombosis or pulmonary embolism, patients with low factor V activity (<150 IU/dL) had higher mortality (30%) than those with higher factor V activity (12% mortality) as the disease progressed [34]. Additionally, Stefely and researchers analyzed the abnormal activated partial thromboplastin time (aPTT) waveform, which is a sign of disseminated intravascular coagulation (DIC). Patients with lowered factor V had an abnormal waveform slope, which shows that having factor V under 150 IU/dL could be an early marker for a DIC reaction leading to coagulopathy [34]. However, as a commentary to the Stefely et al. research, Escher and colleagues noted that due to normal platelet levels and high fibrinogen levels, the signs of DIC were not of a classical pathway but rather were a DIC-like response [35]. Thus, it is suggested that monitoring for fibrinogen, aPTT, factor V (<150 IU/dL), and factor VIII (<200 IU/dL) can prevent increased mortality [34]. Stefely et al. also noted that there was a sex link with factor V. Men tend to have higher factor V with increased SARS-CoV-2 viral load, whereas females tend to have lower factor V with increased SARS-CoV-2 viral load [34]. Although it is a weak correlation with females and factor V, this further complicates using factor V as a diagnostic marker and warrants further investigation. Anti-phospholipid antibodies were also found in 10 patients, among which 40% developed cerebral infarction events. Although there is not enough data to suggest a direct conclusion, the combination of anticoagulants, elevated factor VIII, and presence of anti-phospholipid antibodies could indicate increased coagulopathy in COVID-19 patients [33]. Factor X was speculated to also be involved in COVID-19 related coagulopathy due to its interaction with factor V, however, surprisingly, this was unaltered in COVID-19 (mean 106 IU/dL) [34]. Although factor V, factor VII, factor VIII, and VWF are increased in initial SARS-CoV-2 infection, which generally indicates increased complications, factor V was seen to increase then decrease, which can complicate its use in the prediction of COVID-19-associated complications. 

In alignment to D-dimer, the other coagulation markers and thrombomodulin, prothrombin time (PT) were also elevated in patients with more severe COVID-19, which indicates coagulopathy (Table 3) [28,36,37]. PT and D-dimer positively correlated with the increase in hospital complications using the APACHE II (Acute Physiology and Chronic Health Evaluation II), SOFA (Sequential Organ Failure Assessment), and qSOFA (quick Sepsis Related Organ Failure Assessment) scores to measure mortality and infection in patients admitted to the hospital and the ICU [28]. In a study by Long and colleagues, elevated levels of D-dimer, PT, and aPTT with lower levels of fibrinogen indicated that the coagulopathy seen in COVID-19 patients (n = 115) was caused by a high coagulating state followed by a fibrinolytic state due to the increased consumption of coagulation factors [36]. This is why D-dimer and other fibrinogen degradation products are elevated, whereas a lower factor V can indicate increased mortality [38]. 

Another studied indicator of disease severity was high-sensitivity cardiac troponin-1 (hs-cTn1) or troponin that indicates myocardial infarction when elevated (Table 3). Severe cardiovascular disease patients with COVID-19 had elevated troponin when compared with non-severe patients with COVID-19 [39]. Because troponin indicates cardiac injury and virus-related myocardial injury, through hypoxia and cytokine storm, elevated troponin can be an indicator for severe COVID-19. Patients diagnosed with COVID-19 who develop pneumonia and had elevated hs-cTn1 were associated with having right ventricular dilation and dysfunction [40]. D-dimer and c-reactive protein (CRP) had a better association with right ventricular dysfunction when compared to hs-cTn1. As a result of right ventricular dysfunction, often the left ventricle becomes hyperdynamic to compensate for dysfunction. This indicates that troponin can be used to monitor for cardiovascular risk with COVID-19, although D-dimer is better [39]. 

### 3.2. Inflammation, Immune Response, and Cytokine Storm

Published studies on COVID-19 also looked at many immunological factors because SARS-CoV-2 is a virus and, therefore, inflammation and an immune activation are to be expected. IgM memory B cells can rapidly differentiate into plasma cells and play a major role in early inflammatory responses, including those caused by infections [22]. One of the primary sources of those cells is the spleen, where patients without a spleen or with dysfunctional spleen have a lower number of circulating IgM memory B cells, which is associated with worse health-related outcomes, including greater risk of developing serious infections or overwhelming post-splenectomy infections [23]. Reports suggested that IgM memory B cells are commonly depleted in COVID-19 patients, which correlates with increased mortality and superimposed infections [41].

Generally, patients with COVID-19 had elevated lactate dehydrogenase (LDH) and CRP, which indicates organ damage and inflammation (Table 3). Elevated LDH had an OR of 2.85, whereas elevated CRP had an OR of 2.38 [42]. These factors along with D-dimer and bilateral pulmonary infiltrates were the most important factors for ICU admission and predicting mortality with COVID-19. As for hospitalization, the most important factors were lymphopenia (OR = 3.48) and CRP (OR = 3.27) [42]. IL-6 was another immunological factor that was elevated in COVID-19 patients, with severe COVID-19 having more elevation than in non-severe COVID-19 patients. Gao et al. demonstrated in 43 patients that the optimal threshold of IL-6 was 24.3 μg/L and the area under receiver operator characteristic curve of IL-6 was 0.795 μg/L [43]. The value of the area under receiver operator characteristic curve and D-dimer worked together in predicting the severity of COVID-19 with 93.3% sensitivity in tandem testing and 96.4% sensitivity in parallel testing [43]. This once again shows that IL-6 and D-dimer are both important diagnostic factors in predicting the severity of SARS-CoV-2 infection in patients. IL-6 was also correlated with fibrinogen levels that indicated an interplay between coagulation and inflammation. IL-6 activates tissue factor gene expression in endothelial cells and monocytes, which causes an increase in fibrinogen synthesis. In addition to fibrinogen synthesis and platelet production, IL-6 does not cause fibrinolysis that causes coagulopathy in COVID-19 patients [29]. Yang et al. also showed that similar to IL-6, an increase in IL-2, IL-10, and INF-γ correlated with disease severity [44]. Together, these studies suggest that SARS-CoV-2 infections increase the production of cytokines. 

SARS-CoV-2 might cause decreased lymphocyte subset cells, particularly in T cell counts, which would further induce pro-inflammatory response and cytokine storm and changes in cytokine and chemokine levels, which might be independent predictors for the poor outcome of COVID-19 [45]. In relation to cytokine production, SARS-CoV-2 was also shown to alter the presence of immune cells. Generally, patients with COVID-19 requiring hospital admission showed signs of lymphopenia [46]. In addition, patients who had severe COVID-19 when compared with less severe COVID-19 had significantly lowered lymphocytes [39]. Lymphocyte migration accompanied by macrophages can lead to interstitial damage, which can affect gas exchange, leading to the hypoxemia and dyspnea seen in COVID-19. As a result of these interactions, the neutrophil–lymphocyte ratio has been proposed to be a possible marker of disease severity, with higher ratios showing a greater risk for deterioration. A relative lymphocyte decline would thus be an indirect marker of underlying lung inflammation [46]. Zhang and researchers also demonstrated that diabetic patients (n = 166) with COVID-19 had higher leukocytes and neutrophils counts with lowered lymphocytes and eosinophils [47]. In addition, due to the pre-existing decrease in T-cell, neutrophil, macrophage, and lymphocyte function seen in diabetic patients, this showed that diabetic patients were more susceptible to infection and worsened outcomes. Together with the altered immune cell presence and the increase in cytokines, this put COVID-19 patients at risk for developing cytokine storm. However, in a recent study it was shown that patients who had severe COVID-19 and survived had T-cell counts and cytokine levels that returned to that of mild COVID-19, which suggests that resolution is possible with concurrent treatment [38].

During the cytokine cascade, an excessive immune response produces injury to multiple organs including lung tissue. This damage can cause dyspnea, which is one of the premonitory symptoms of respiratory complications such as acute respiratory failure. Because blood ferritin levels can elevate with cytokine storm, it is recommended to monitor ferritin levels in COVID-19 patients [48]. SARS-CoV-2 could also damage lymphocytes, particularly T-cells, which can lead to lymphocytopenia [49]. The consequences of these interactions lead to the formation of a cytokine storm, which can cause a series of damaging immune responses [50]. With the presence of cytokines (TNF-1 and IL-6) and chemokines (IL-8), patients with severe COVID-19 had a higher incidence of structural damage in the body. These damages included vasculature (multiple micro-thrombosis), pulmonary (pulmonary fibrosis), nervous (headache and loss of smell and taste), and cardiovascular (myocardial infarction) damages [51,52]. 

Interestingly, in a study by Sun and colleagues of calcium, parathyroid hormone (PTH), and vitamin D concentrations, lowered serum calcium levels led to increased organ injury and septic shock and worsened 28-day mortality in patients (n = 241) with COVID-19 [53]. Although calcium might be another parameter suggestive in predicting COVID-19 severity, further studies are needed to show the effectiveness of calcium to determine COVID-19 outcomes. 

### 3.3. Other Risk Factors, Comorbid Disease, and Organ Failure

In patients with SARS-CoV-2 infection who are diagnosed with hypertension compared with patients who do not have hypertension, the need for noninvasive mechanical ventilation and mortality was higher in patients with hypertension. Although all patients with COVID-19 had increased lactate dehydrogenase, eosinophil sedimentation rate, CRP, serum ferritin, IL-6, and D-dimer, which are all associated with infection and cardiac damage, patients with hypertension had higher neutrophils and neutrophil–lymphocyte ratios, ALT, creatinine, and fibrinogen compared with non-hypertensive patients, indicating that COVID-19 patients with hypertension tend to show more severe inflammation and organ damage than in patients without hypertension (Table 3) [54]. This is speculated to be a result of an imbalanced renin–angiotensin system found in hypertensive patients. Excessive inflammatory mediators and cytokines can lead to an overactivated NADH/NADPH oxidase, leading to increased cell damage and vasoconstriction. This is often presented as lung damage, resulting in many of the respiratory symptoms seen in COVID-19 patients. 

In patients who are diagnosed with diabetes (fasting blood glucose > 126 mg/dL and HbA1c > 6.5), secondary hyperglycemia (fasting blood glucose > 126 mg/dL and HbA1c < 6.5), or not diabetic (fasting blood glucose < 126 mg/dL)), increased hospital mortality was seen with patients who had a new onset of hyperglycemia when compared with individuals with a history of diabetes or normoglycemia [47]. Although this was surprising, diabetes has been associated with weakened immune response and a higher risk for bacterial co-infections, and as such, it is recommended that patients who are prediabetic or recently diagnosed with diabetes monitor blood glucose in hopes to prevent worsened outcomes with COVID-19. 

In healthy patients who are infected with SARS-CoV-2, ischemic stroke is a complication of COVID-19 due to coagulopathy and DIC-like symptoms. A study published by Yaghi et al. indicated that patients (n = 32) who had a stroke with COVID-19 were generally healthier and younger than patients in the stroke control group (n = 32) who were COVID-19 negative [55]. Thus, therapeutic anticoagulation in patients with higher D-dimer is beneficial in preventing mortality. Similarly, monitoring for D-dimer is more important than ever in these populations.

In terms of organ damage, increased albumin, BUN, aspartate transaminase (AST), and alanine aminotransferase (ALT) seen in patients with COVID-19 confirm that organ damage was most likely present [26,42]. Using the elevated factors, acute kidney injury is most likely caused by the virus invasion that can lead to a decrease in oxygen perfusion and shock [26]. In addition, the combination of cytokines and chemokine-induced cytokine storm can also contribute to the elevation in these parameters, indicating a more severe disease with multiple organ involvement. This shows that not only does COVID-19 cause a dysregulation in coagulation and immune functions but that the dysregulation of these systems can lead to permanent organ damage. 

The combination of these factors indicates that patients with underlining disease such as hypertension, uncontrolled or recent onset of diabetes, obesity, ischemic stroke, or thrombosis with SARS-CoV-2 infection and diagnosed with COVID-19 are at increased risk of mortality. Therefore, D-dimer, blood glucose, and IL-6 should be monitored to prevent COVID-19-associated complications.

### 3.4. RT-PCR Sampling Site, Methodology, and Testing Capacity

Although RT-PCR has become the most used testing method for patients who are suspected of having a SARS-CoV-2 infection, the best strategy for sample collection is another area that requires investigation. This is important because samples with low viral load can lead to a false negative result. In a study by Ye et al., RT-PCR samples (n = 91) were collected using a tongue swab and a pharyngeal swab [56]. The study showed that the sensitivity of the test was dependent on the experience and technique of the professional collecting the sample. The results from a single experienced individual and multiple experienced individuals showed a difference in results, indicating a need for standardization. When multiple experienced individuals sampled the different collection sites, there was a greater detection by the tongue swab when compared to the pharynx swab, although not significant (35.6% vs. 33.3%) [56]. In a study by Yang et al. (n = 866), different sample sites were separated into three groups based on the collection time in days after the onset of symptoms (0–17 d, 8–14 d, and > 15 d) [57]. Samples collected from the sputum in the 0 to 7 days group had the highest positive rate in mild (82.2%) and severe (88.9%) cases, followed by nasal swabs (72.1% and 73.3%, respectively), and then pharyngeal swabs (61.3% and 60.0%, respectively). Bronchoalveolar lavage collected from days 8 to 14 in severe cases showed a 100% positive result. Sputum collected from the upper respiratory tract during days 8 to 14 showed a high rate of positive results in mild and severe cases that was greater than the nasal and pharyngeal swab rates. The positive rate of pharyngeal swabs was only 29.6% in mild cases and 50% in severe cases. Although these results suggest the use of sputum for the highest detection rate, this research has yet to be peer-reviewed, and these results need to be confirmed. Similarly, Woelfel et al. stated that the best detection site with the highest virus load was from throat- and lung-derived samples and not alternative sites such as stool, blood, or urine [58]. The tested patients did not present with severe COVID-19, and thus the presence of the virus in the stool, blood, or urine might still occur in more severe cases but be undetected in mild COVID-19. Although the specific location is still being investigated, in accordance with the currented recommended sampling sites, Yang and Woelfel’s studies demonstrated that routine testing for mild COVID-19 is completed either though oropharyngeal or nasopharyngeal methods [20,21]. 

In a study by To and colleagues, nasopharyngeal sampling by a healthcare professional was compared to saliva sampling by the patient [59]. Using RT-PCR, high sensitivity was obtained from samples that were collected by the patient. Thus, self-sampling can also be a viable option to reduce and prevent additional risks with infection during collection. Although these results need to be further investigated, self-sampling can be an option for individuals who have severe COVID-19 due to elevated viral load and to minimize the risk of infection during collection. This study also suggests that the results from Yang et al. with regards to increased detection in sputum sample can be beneficial in preventing false negatives [57,59]. 

The current reference test is the RT-PCR or virological test and consists of detecting the genetic material of SARS-CoV-2 by means of a nasopharyngeal test, allowing clinicians to know if, at the time when the test was performed, the patient is infected with the virus. This test can also be performed using a salivary sample, which is simpler and less unpleasant, but saliva is less reliable in detecting the virus. The serologic tests consist of a blood test in order to know if the patient developed antibodies against SARS-CoV-2 and, therefore, if they were infected with coronavirus at some point. There is also a quick version of these, which only requires a drop of blood and can be practiced by a health professional or a pharmacist, but they are considered indicative, and it is recommended to confirm the result in a laboratory [41].

Nonetheless, the National Health Commission of China suggests collecting samples from the upper respiratory tract (nasopharyngeal or pharyngeal swabs) and the lower respiratory tract (deep-cough sputum, alveolar lavage fluids, bronchial lavage fluids, and respiratory tract extracts) in the acute phase of infection [20]. The lower respiratory tract is preferred in patients who develop signs and symptoms of severe COVID-19. Although stool, blood, and serum samples are in the protocol, these samples were only suggested on an “as needed” basis for clinical needs. Knowing the limits of RT-PCR and the aforementioned standardization of sampling can help prevent false negatives during COVID-19 diagnosis, which can prevent the spread of SARS-CoV-2.

The reliability of the tests is defined by the specificity, that is, the ability to correctly detect SARS-CoV-2 and nothing else, and the sensitivity when detecting the virus even if there is only a very high quantity. The specificity rate of RT-PCR is 99%, so false positives are very rare. RT-PCR also detects very small concentrations, which makes it unlikely to miss infected people. However, in situ, the sensitivity of PCRs depends on how the test is performed, and therefore, false negatives can occur when the sample is not drawn correctly. As mentioned earlier, the time of sampling during the course of infection can also impact the sensitivity and specificity. This justifies the need for repeated testing. Paradoxically, the extreme sensitivity could also be a drawback for patients who test positive but have viral loads too low to be clinically relevant (ability to infect other individuals). However, it is not yet known with certainty the threshold viral load to which one could still be deemed as contagious or be able to readily spread the virus [41,45]. In areas where the rates of SARS-CoV-2 infection are much lower than the number of individuals who test for SARS-CoV-2, pooled saliva testing with RT-PCR can increase the testing capacity. However pooled testing does have its own drawbacks due to the dilution of positive samples that can shift weakly positive samples below the limit of detection. Watkins and colleagues demonstrated that pooling 5, 10, and 20 samples for quantitative RT-PCR (RT-qPCR) led to a reduction in sensitivity by 7.41%, 11.11%, and 14.81%, respectively [60]. As a result of a decrease in sensitivity, in populations where the prevalence of SARS-CoV-2 is greater than 3%, testing samples in pools of five or less can lead to the least overall number of tests, and in populations where the prevalence of SARS-CoV-2 is less than 1%, pools as large as 20 can be more beneficial and more supportive of ongoing preventative screening [60]. One way to bypass the decrease in sensitivity is to retest all positive pooled samples individually, however, in populations where the prevalence is high, this can use up more resources than traditional testing methods. This suggests that pooled testing should only be used in populations where infections are low. Pooled testing, although it increases the risk for false negatives, can be beneficial as more individuals seek validation from a negative COVID-19 test when they have symptoms of the annual winter flu, which often overlap with COVID-19 symptoms.

### 3.5. Chest CT-Scan 

In patients who have severe COVID-19 or where RT-PCR is not available, a chest CT-scan is a valid alternative. Chest radiography is not a first-line test for COVID-19 because of its limited sensitivity in finding ground-glass opacities in SARS-CoV-2 infection [61,62]. However, studies by Kanne et al. and Zu et al. demonstrated that CT-scans could be used in detecting SARS-CoV-2 induced damage in the lungs with high sensitivity but limited specificity [62,63]. Comparing patients with mild to moderate COVID-19 and severe COVID-19 (n = 82 in total), XIe et al. found that CT-scans detected ground-glass opacity in 66.1% of these patients, with bilateral involvement in 77.4% and gastrointestinal abnormalities in 4.8% [39]. 

Additionally, in some studies, the use of a CT-scan has shown some inconsistency between laboratory results and symptoms. Up to half of COVID-19 patients who underwent a CT-scan that indicated no infection had developed symptoms two days earlier. This discrepancy indicates that CT-scans cannot rule out SARS-CoV-2 infections [63]. As a result, in patients showing symptoms of SARS-CoV-2 who have a healthy CT-scan and a negative RT-PCR, additional laboratory tests (such as D-dimer and IL-6) and quarantine are recommended [52]. The use of CT-scans to rule out infection is not recommended but should be corroborated by other COVID-19 tests.

Although D-dimer can predict the severity of COVID-19, CT imaging was able to show the progression of disease and the DIC-like reaction [36]. Additionally, the use of CT angiography allowed the identification of pulmonary embolism after the hypercoagulation state, which can help prevent mortality [36]. Therefore, the use of CT-scan along with other marks of poor COVID-19 prognosis (D-dimer, IL-6, cytokine storm, coagulopathy, comorbid conditions such as hypertension, obesity, diabetes, and advanced age) can help identify complications in patients with SARS-CoV-2 infection. 

### 3.6. Serological Testing

In addition to RT-PCR and CT-scans, another method for detecting viral particles is to use serological methods. Multiple studies evaluated the diagnostic accuracy of serological test, the accuracy of viral molecule amplification techniques, the detection of viral antigens, or strategies of collecting samples for viral replication test (Table 4) [46,64,65,66,67,68,69,70]. These studies were conducted in the laboratory or at the point of care using techniques such as enzyme-linked immunosorbent assay (ELISA), chemiluminescence immunoassay (CLIA), colloidal gold-immunochromatographic assay (GICA), lateral flow immunoassay (LFIA), or magnetic chemiluminescence enzyme immunoassay (MCLIA) for the detection of IgA, IgM, or IgG, either alone or in combination. The diagnostic accuracy of these tests was assessed against the gold standard, RT-PCR, or in relation to the diagnostic criteria set forth by the World Health Organization or the CDC [71,72,73]. 

Most of the studies that evaluated the accuracy of serological testing for SARS-CoV-2 showed high sensitivity and high specificity. However, the performance of the test varied greatly depending on the patient’s disease progression. Unfortunately, this was hard to determine, as only two studies, Guo and colleagues and Li and colleagues, indicated the time between symptom onset and when the test was reported (39 days and 33 days, respectively) [46,65]. The greatest limitation of these test is the window between SARS-CoV-2 infection and antibody production at detectable levels, which can lead to false negatives when the tests are applied too early in infection. Guo and colleagues showed that the median days for the minimum detection of IgA and IgM was 5 days with 77.9% of samples showing a positive result [46]. The median for IgG detection occurred around day 14 [46]. The sensitivity rates found in this study (93.3 for IgA and 90.4 for IgM) should be interpreted with caution, as variability is possible. 

The potential advantages of serological testing are that it can be administered in a point of care setting, which allows for increased testing capacity. However, even with high sensitivity (all results above 86%) and specificity (all results above 95%), indicating a low probability of false positives, cross-reactivity with another coronavirus such as SARS-CoV-1 or MERS-CoV still cannot be overlooked. The sensitivity of the serological tests is comparable to the sensitivity of RT-PCR, which suggests its use in screening for SARS-CoV-2. Additionally, Li and colleagues demonstrated that the accuracy of combined IgM and IgG tests showed consistency between samples collected peripherally, with a fingertip puncture, and in venous puncture, which shows that these tests could be minimally invasive [65]. Unfortunately, most serological tests require the use of whole blood, serum, and plasma samples for the detection of SARS-CoV-2 antibodies. In addition, due to the risk of false positives in early stages of infection, these interpretations must be completed by a doctor when patients present with signs and symptoms of SARS-CoV-2 infection. Therefore, even serological testing requires confirmation from another established test, and this limits its use.

With vaccines now being given to prevent the spread of SARS-CoV-2 infection, it is not certain if serological tests can detect antibodies protecting against reinfection or for how long one would be considered immune. Instead, they are most useful for determining the proportion of infected individuals [41,45].

**Table 3:** A summary of different parameters and their combinations in COVID-19 positive patients and their outcomes, complications, and risk factors.

Measured Parameter(s)	Results	Ref.
VWF	• Patients in ICU having even higher VWF than hospitalized patients	[32]
↑Thrombomodulin	• Increased in-hospital mortality	[32]
↑D-dimer	• >1 μg/mL led to increased bleeding or thrombosis	[10]
	• >1.56 μg/mL led to increased mortality	[26]
	• Pediatric patients in the ICU had the highest D-dimer followed by patients in the hospital and outpatient individuals	[27]
	• Increased coagulation disorders and thrombi formation	[28,29]
	• Formed clots that were firmer than in COVID-19 negative patients	[29]
	• >1.5 μg/mL had 85% sensitivity, 88.5% specificity detecting VTE	[30]
	• Increased risk for thrombosis	[31]
↑D-dimer, ↑CRP	• More correlated with RV dysfunction compared to ↑hs-cTn1	[40]
↑D-dimer, ↑PT, ↑aPTT, ↓fibrinogen	• High coagulating state followed by fibrinolytic state due to consumption of coagulation factors	[36,37,38]
Factor V	• Factor V was elevated (>150 IU/dL) in initial SARS-CoV-2 infection	[34]
	• Low factor V (>150 IU/dL) correlated with higher mortality	[34]
	• Factor V is positively correlated with viral load in men and weakly negatively correlated with viral load in females	[34]
↓Factor V, aPTT	• Lowered factor V led to abnormal aPTT waveform, showing DIC reaction leading to coagulopathy.	[34]
	• The DIC-like reaction seen in COVID-19 is through a non-classical pathway that leads to a DIC-like response	[35]
↓Factor V, ↓ Factor VII	• In terminal stage patients	[34]
↑Factor VII	• >200 IU/dL can increase mortality	[34]
↑Factor VIII	• Seen in COVID-19 positive patients with a 307% increase showing coagulopathy	[32,34]
Factor X	• Factor X is unaltered in COVID-19	[34]
↑PT	• Correlated with complications predicted by APACHE II, SOFA, and qSOFA scores	[28]
↑Anti-phospholipid antibodies	• Indicates increase coagulopathy	[33]
↑hs-cTn1	• In severe COVID-19, indicates myocardial infarction and RV dysfunction	[39]
↑LDH, ↑CRP, ↑D-dimer, ↑bilateral pulmonary infiltrates	• Determining ICU admission and predicting mortality	[42]
↑IL-6	• Severe COVID-19 patients had a greater increase than non-severe COVID-19 patients	[42]
	• Optimal threshold is 0.795 μg/L	[43]
↑IL-6, ↑fibrinogen	• IL-6 and fibrinogen can cause coagulopathy	[29]
↑IL-6, ↑IL-2, ↑IL-10, ↑INF-γ	• Predicts disease severity	[44]
↑IL-6, ↑TNF-1, ↑IL-8	• Increased organ damage, vasculature (multiple micro-thrombosis), pulmonary (pulmonary fibrosis), nervous (headache and loss of smell and taste), and cardiovascular (myocardial infarction)	[51,52]
↓Lymphocytes	• Hospitalization, increased COVID-19 severity • Underlying lung inflammation	[39,46]
↓Lymphocytes, ↑CRP	• Predicted hospitalization	[42]
↑Leukocytes, ↑neutrophils, ↓lymphocytes, ↓eosinophils	• Diabetic patients at increased risk for infection and worsened outcomes	[47]
↑Neutrophil, ↑neutrophil-lymphocyte ratio, ↑ALT, ↑creatinine, ↑fibrinogen	• Increased inflammation and organ damage in hypertensive patients	[54]
↓Calcium	• Increase organ injury, septic shock, and worsened 28-day mortality	[53]
↑BUN, ↑AST, ↑ALT	• Organ damage present with SARS-CoV-2 infection, most likely acute kidney injury	[26,42]

APACHE II—acute physiology and chronic health evaluation II, aPTT—activated partial thromboplastin time, ALT—alanine transaminase, AST—aspartate transaminase, BUN—blood urea nitrogen, CRP—c-reactive protein, DIC—disseminated intravascular coagulation, hs-cTn1—high-sensitivity cardiac troponin-1, ICU—intensive care unit, IL—interleukin, INF-γ—interferon gamma, LDH—lactate dehydrogenase, PT—prothrombin time, qSOFA—quick sepsis related organ failure assessment, RV—right ventricular, SOFA—sequential organ failure assessment, VTE—venous thromboembolism, VWF—von Willebrand factor, ↑—increased/elevated, ↓—decreased.

**Table 4:** Comparing sensitivity and specificity between different studies that looked at serological tests.

Study	Experimental Test	Control Test	Results		Ref.
			Sensitivity (%)	Specificity (%)	
Profiling early humoral response to diagnose novel coronavirus disease (COVID-19)	ELISA (IgA, IgM)	RT-PCR, patient symptoms, CT-scan	IgA—93.3I gM—90.4	IgA—100 IgM—100	[46]
Diagnostic value and dynamic variance of serum antibody in coronavirus disease 2019	CLIA (IgG, IgM)	RT-PCR	IgG—88.9 IgM—48.1	IgG—90.9 IgM—100	[64]
Development and clinical application of a rapid IgM–IgG combined antibody test for SARS-CoV-2 infection diagnosis	Immunoassay (IgG, IgM), LFIA	RT-PCR	Overall—88.66	Overall—90.63	[65]
A peptide-based magnetic chemiluminescence enzyme immunoassay for serological diagnosis of coronavirus disease 2019	MCLIA (IgG, IgM, IgG-IgM)	RT-PCR	IgG—71.4I gM—57.2 IgG-IgM—81.5	IgG—100 IgM—100 IgG-IgM—100	[64]
Evaluation of enzyme-linked immunoassay and colloidal gold- immunochromatographic assay kit for detection of novel coronavirus (SARS-Cov-2) causing an outbreak of pneumonia (COVID-19)	ELISA or GICA (IgG, IgM, IgG-IgM)	WHO diagnostic criteria	ELISA(IgG)—82.5 ELISA(IgM)—44.4 ELISA(IgG-IgM)—87.3 GICA(IgG)—81.3 GICA(IgM)—57.1 GICA(IgG-IgM)—82.4	ELISA(IgG)—100 ELISA(IgM)—100 GICA(IgG)—100 GICA(IgM)—100	[67]
Serological diagnostic kit of SARS-CoV-2 antibodies using CHO-expressed full-length SARS-CoV-2 S1 proteins	ELISA (IgM, IgG)	Control group *	Overall—99.7	Overall—97.5	[68]
Performance of VivaDiag COVID-19 IgM/IgG Rapid Test is inadequate for diagnosis of COVID-19 in acute patients referring to emergency room department	LFIA	RT-PCR	Inpatient—83.3 Outpatient—18.4	Inpatient—100 Outpatient—91.7	[69]
Evaluation of nine commercial SARS-CoV-2 immunoassays	ELISA, LFIA	RT-PCR	ELISA—65–90 LFIA—83–90	ELISA—93–100 LFIA—100	[70]

* Diagnosis methods were not reported. CLIA—chemiluminescence immunoassay, ELISA—enzyme-linked immunosorbent assay, GICA—colloidal gold-immunochromatographic assay, IgA—immunoglobulin A, IgG—immunoglobulin G, IgM—immunoglobulin M, LFIA—lateral flow immunoassay, MCLIA—magnetic chemiluminescence enzyme immunoassay, RT-PCR—reverse transcription polymerase chain reaction.

## 4. Alternative Testing and Future Perspectives

Although RT-PCR is the gold standard, analyzing RT-PCR results takes time and often requires several days to send the results to patients. Given the saturation of laboratories, public authorities seek to expand the range of tests, in order to effectively detect potentially infected individuals, either at an airport or in a medical consultation. Thus, rapid antigen tests make it possible to detect an infection in between 15 and 30 min. Like RT-PCR, sampling is completed through the nasopharyngeal route but instead of detecting the SARS-CoV-2 genome, they look for viral proteins, a simpler and faster system that does not require the intervention of a laboratory. Currently, rapid antigen tests require qualified personnel, and they are also considered less reliable than PCRs. For healthcare authorities, the question is to determine if its level of reliability is acceptable given the need in some areas to diagnose such a large number of people. On the other hand, doctors and laboratories are asking for a better selection of individuals who undergo the tests, prioritizing the most vulnerable and those with symptoms to be able to effectively complete contact tracing and prevent further spread of COVID-19 [41,45].

In addition to rapid antigen testing, reverse transcriptase loop-mediated isothermal amplification (RT-LAMP), mass spectrometry, and clustered regularly interspaced short palindromic repeats (CRISPR) technology are also alternatives that can increase turnover time and increase the testing capabilities for SARS-CoV-2. RT-LAMP, which amplifies viral particles, has good sensitivity (97.5%) and specificity (99.7%) when compared to RT-PCR. Even without RNA isolation, RT-LAMP showed excellent sensitivity (99.5%) but lowered sensitivity (86%) compared to RNA-isolated RT-LAMP [74]. RT-LAMP was demonstrated to be faster, simpler, and more cost effective when compared to RT-PCR. Nonetheless, due to the limitation of sampling location, which can alter the overall viral load, and how RT-LAMP would show in patients who are shedding SARS-CoV-2 virus, the use of RT-LAMP warrants further studies.

Another alternative to increased testing capacity is to use mass spectrometry. Although RT-PCR is the gold standard, viral detection with mass spectrometry can lead to a faster turnover time and thus a higher testing capacity. Mass spectrometry is also a cost-effective alternative to RT-PCR [75]. Although the hands-on-time with running a RT-PCR and mass spectrometry is similar, both methods isolated and amplified viral RNA from oropharyngeal and nasopharyngeal swabs with no variation. However, because only 22 known positive and 22 known negative samples were used, the results from this study warrant an upscaled investigation [75]. RT-PCR provides far more reliable results with least possible number of false positive results. Nonetheless, testing with mass spectrometry is a successful proof-of-concept alternative.

A promising future for COVID-19 testing lies in the use of CRISPR technology. First identified by Feng Zhang’s laboratory in 2017, CRISPR-Cas13a, when complementary to a guide RNA, was able to cleave the RNA [76]. Zhang’s protocol included small synthetic RNA molecules that had a fluorescent marker conjugated in the test tube and an inhibitor of the fluorescence flanking each end. Initially, these molecules did not fluoresce (the presence of the inhibitors prevented fluorescence), but as the activated Cas13a cleaved the RNA, the fluorescent markers detached from the inhibitors, which were measured. This novel system for diagnosing the presence of a specific RNA in a mixture was coined Specific High-sensitivity Enzymatic Reporter un-LOCKing (SHERLOCK). The adaptation of SHERLOCK to detect the SARS-CoV-2 virus within 1 h in a concentration as low as 10 copies/μL suggests a very bright future in COVID-19 diagnosis [76]. However, SHERLOCK is still under validation and approval by the FDA for its use in hospitals, thus, follow-up studies are required. 

Similar to SHERLOCK, another diagnostic tool that uses CRISPR-Cas12a as developed by Ding et al. in 2018 works to cut single-stranded DNA using a guide RNA [77]. When used in combination with the protocol developed by Zhang’s laboratory, it can also be adapted to detect SARS-CoV-2 [76,77]. The system, DNA endonuclease targeted CRISPR trans reporter (DETECTR), when used in RNA viruses such as SARS-CoV-2 must first convert its DNA using a reverse transcriptase cycle [78]. Once the virus genome is converted into DNA, it can then be detected with the corresponding RNA guide to produce a fluorescent signal or a chemical signal. Together, the diagnostic tool DETECTR shares similar proof-of-concept sensitivity with SHERLOCK [76,78]. Currently, the FDA is also validating the use of DETECTR in hospitals, and follow-up studies are also required.

Finally, a third developing CRISPR technology for detecting SARS-CoV-2 is named Combinatorial Array Reactions for Multiplexed Evaluation of Nucleic acids with Cas-13 (CARMEN-Cas13) [79]. CARMEN functions as a combination of SHERLOCK with microfluidic technology (nano-drops) on a microchip that carries out thousands of simultaneous detection reactions (massively multiplexed nucleic acid detection). The versatility of CARMEN in detecting COVID-19 in more than a thousand samples simultaneously can address the many issues discussed herein with regards to limited testing capacity.

## 5. Different COVID-19 Diagnostic Tests, Their Advantages and Limitations

For the different COVID-19 diagnostic test and their pros and cons, see Table 5.

### 5.1. Reverse-Transcription Polymerase Chain Reaction (RT-PCR)

Pros: (a) RT-PCR is an established, robust, and well documented technique, and (b) RT-PCR can detect current infections.

Cons: (a) RT-PCR can only detect current infections and takes some time, and (b) it cannot provide information on symptoms.

### 5.2. Loop-Mediated Isothermal Amplification (LAMP)

Pros: (a) LAMP is a quick (2–3 h) and cheap technique, and (b) LAMP can detect current infections of disease. 

Cons: (a) LAMP cannot provide information on symptoms and does not tell staff if a patient has been previously infected with the virus or if a patient has any immunity to the virus.

### 5.3. Lateral Flow

Pros: (a) It is extremely quick per patient, giving results in just 15 min.

Cons: (a) It is new technique, and the evidence for its accuracy in coronavirus diagnosis is still being evaluated, and (b) it is more expensive and time-consuming for large-batch testing.

### 5.4. Enzyme-Linked Immunosorbent Assay (ELISA)

Pros: (a) ELISA is scalable laboratory technique and gives results within 1 to 3 h.

Cons: (a) ELISA tests are not yet well established and validated for SARS-CoV-2 COVID-19 testing, but it is progressing.

## 6. Conclusions

COVID-19 diagnosis is integral to the subsequent treatment and management of patients who are infected with SARS-CoV-2. The identification of increased D-dimer (>1–2 μg/mL), increased thrombomodulin (>3.26 ng/mL), initial increase in factor V (>150 IU/dL) followed by late-stage low factor V (<150 IU/dL) or low factor VII (<200 IU/dL), and elevated IL-6 (>0.795 μg/L), LDH, and CRP have been associated with coagulopathy (such as DIC-like reactions) and cytokine storm correlated with increased mortality in patients with COVID-19 or COVID-19-associated pneumonia. Patients with risk factors such as recently diagnosed diabetes (fasting blood glucose > 126 mg/dL and HbA1c < 6.5), hypertension, obesity, and old age are also at increased risk for COVID-19 complications. Patients with severe COVID-19 had elevated BUN, AST, and ALT, which indicated AKI and other viral-induced organ damage. COVID-19 is currently being sampled using sputum, oropharyngeal, or nasopharyngeal methods in initial COVID-19 screening and tested using the gold standard testing method, RT-PCR. In cases where RT-PCR is not available or in patients with severe COVID-19, CT-scans can be used with signs and symptoms of SARS-CoV-2 infection in the presence of a doctor to diagnose COVID-19. Serological tests such as measuring IgG and IgM are also possible due to the lower turnover of RT-PCR, however, the time from symptom onset to test should be considered for sensitivity and selectivity. Due to the low-turnover rate and increased demand for COVID-19 testing, pooled RT-PCR is possible for populations where infection is less than 1%. Alternative methods such as RT-LAMP, mass spectroscopy, and CRISPR technology (SHERLOCK, DETECTR, and CARMEN) that are in development can be used down the line to help increase turnover time and testing capacity. The proper diagnosis of COVID-19 is critical in managing patients with SARS-CoV-2 infection and preventing COVID-19-associated complications.

## Figures and Tables

**Table 1:** Pneumonia severity index (PSI).

PSI Score	Mortality Risk (%)	Treatment Strategy
≤70	0.6–0.7	Outpatient
70–90	0.9–2.8	Outpatient
91–130	8.5–9.3	Brief Hospitalization
>130	27–31.1	Intensive Care Unit (ICU)

**Table 2:** CURB-65 criteria: Each item in the CURB-65 criteria scores 1 point. Hospital admission is recommended if the patient has a total score greater than or equal to 1 point.

CURB-65	Criterion
C	Acute Confusion
U	Urea > 18 mg/dL
R	Respiratory Rate > 30 RPM
B	Systolic blood pressure ≤ 90 mmHg or diastolic blood pressure ≤ 60 mmHg
65	Age ≥ 65

RPM—respirations per minute, mmHg—millimeters of mercury.

**Table 5:** Profiles of different diagnostic tests and their advantages and limitations.

Technology	Molecule Tested	LB or POC	Time to Results	Typical Sample Site	Number of Samples Tested Per Batch
RT-PCR	viral RNA	LB	3–4 h	Nasopharyngeal swab, sputum	Up to 96 samples (lab based)
LAMP	viral RNA	LB or POC	2–3 h	Nasopharyngeal swab, sputum	96 samples or 1–4 samples at Point of Care.
Lateral Flow	Antibody	POC	15–20 m	Blood for antibody testing	1 patient sample
ELISA	Antibody	LB	1–3 h	Blood for antibody testing	Up to 96 samples

LB = Laboratory Based, POC = Point of Care.

## Abbreviations

ACE2	angiotensin-converting enzyme-2
ALT	alanine aminotransferase
APACHE II	acute physiology and chronic health evaluation II
aPTT	activated partial thromboplastin time
AST	aspartate transaminase
BUN	blood urea nitrogen
CARMEN	combinatorial array reactions for multiplexed evaluation of nucleic acids
CLIA	chemiluminescence immunoassay
CRISPR	clustered regularly interspaced short palindromic repeats
CRP	c-reactive protein
CURB-65	acute confusion, urea concentrations, respiratory rate, blood pressure, age > 65
DETECTR	DNA endonuclease targeted CRISPR trans reporter
DIC	disseminated intravascular coagulation,
ELISA	enzyme-linked immunosorbent assay
GICA	colloidal gold-immunochromatographic assay
HbA1c	hemoglobin A1chs-cTn1—high-sensitivity cardiac troponin-1
IL	interleukin
INF-γ	interferon gamma
LDH	lactate dehydrogenase
LFIA	lateral flow immunoassay
MCLIA	magnetic chemiluminescence enzyme immunoassay
MERS-CoV	Middle East respiratory syndrome coronavirus
NADH/NADPH	nicotinamide adenine dinucleotide/nicotinamide adenine dinucleotide phosphate
PaO_2_/FiO_2_	partial pressure of oxygen in blood over fraction of inhaled oxygen
PSI	pneumonia severity index
PT	prothrombin time
PTH	parathyroid hormone
qSOFA	quick sepsis related organ failure assessment
RT-LAMP	reverse transcriptase loop-mediated isothermal amplification
RT-PCR	reverse transcriptase polymerase chain reaction
RT-qPCR	quantitative RT-PCR
RV	right ventricular
SARS-CoV-2	severe acute respiratory syndrome coronavirus-2
SHERLOCK	specific high-sensitivity enzymatic reporter un-locking
SOFA	sequential organ failure assessment
TNF-1	tumor necrosis factor-1
VTE	venous thromboembolism
VWF	von Willebrand factor
